# Evaluating Ecosystem Services Supply and Demand Dynamics and Ecological Zoning Management in Wuhan, China

**DOI:** 10.3390/ijerph16132332

**Published:** 2019-07-02

**Authors:** Feiyan Chen, Ling Li, Jiqiang Niu, Aiwen Lin, Shiyu Chen, Lin Hao

**Affiliations:** 1 School of Resource and Environmental Science, Wuhan University, Wuhan 430079, China; 2 School of Geographic Sciences, Xinyang Normal University, Xinyang 464000, China; 3 Key Laboratory for Synergistic Prevention of Water and Soil Environmental Pollution, Xinyang Normal University, Xinyang 464000, China

**Keywords:** ecosystem services, ecosystem services supply and demand index, spatial mismatch, Wuhan

## Abstract

The concept of ecosystem services (ES) supply and demand has attracted increasing attention in science and policy making because it effectively links ecosystem services to human well-being. The imbalance of ES supply and demand in urban areas has become a key issue in regional sustainable development. In this context, we calculated ES supply and demand for Wuhan City, China, using the ES supply and demand ratio (ESDR) and the comprehensive ES supply–demand ratio (CESDR) to express the relationship between ES supply and demand. Ecological zoning was proposed according to the spatial differentiation of the ES supply–demand relationship, and policy recommendations are made. The results show that from the perspective of total ES supply and demand, the water yield supply (S_WY_), grain yield supply (S_GY_), and recreation services supply (S_RS_) are greater than the water yield demand (D_WY_), grain yield demand (D_GY_), and recreation services demand (D_RS_), and that the climate regulation supply (S_CR_) is less than the climate regulation demand (D_CR_). From a spatial perspective, there are imbalances and mismatches in ES supply and demand, especially in urban central areas. The values of S_WY_, S_GY_, S_CS_, and S_RS_ per unit area are less than their respective demand values, and the area of mismatch has expanded with the gradual increase of the built-up area. The spatial pattern of ES supply and demand is circular, with the form of “deficit zone–relative equilibrium zone–surplus zone”, which corresponds to “urban central area–near suburbs–distant suburbs and rural areas”.

## 1. Introduction

The term ecosystem services (ES) refers to the various types of well-being that ecosystems provide to humans. They are usually classified into supporting, provisioning, regulating, and cultural services [1,2,3]. The ES supply refers to the products and services provided by an ecosystem for human well-being, and the ES demand is the consumption of products and services provided by the ecosystem [4,5]. The difference in the degree of matching between ES supply and demand reflects, in a sense, the interrelationship between ecosystem services and human well-being [6]. Ecological and environmental problems in a region, especially in urban areas, are fundamentally caused by imbalances or mismatches in ES supply and demand [7]. Therefore, it is important to investigate the types, quantities, dynamic changes, and mutual relationships of ES supply and demand, in order to further develop scientific and rational management methods for the provision of ecosystem services.

Several studies of the assessment and quantitative analysis of regional ES supply and demand have been conducted in recent years [7,8,9]. Burkhard et al. proposed a land cover-based matrix model for assessing the supply and demand of ecosystem services for each type of land cover through an expert assessment methodology [4]. On the basis of environmental quality standards and policy goals, Baró et al. provided environment quality standards to assess ES mismatches, which were then applied to a case study of European cities [10]. Stürck et al. analyzed the role of land-use change in the supply of two regulatory services, flood regulation and climate regulation, in the European Union (EU) for the period of 1900−2000, and they also investigated four plausible scenarios of land-use change up to 2040. The demand for these services was found to increase rapidly, and land-use allocation favoring the supply of regulating services could be seen as a nature-based solution [11]. Zoderer et al. explored the spatial mismatch between the delivery of ES bundles by the ES bundles demand in South Tyrol in the Central Alps on the basis of landscape photographs obtained by local farmers, local inhabitants, and visitors [12]. Schirpke et al. mapped the supply, flow, and demand at the municipality level in the Alpine Space area, then used cluster analysis to analyze the linkages between ES [13]. Wang et al. used the ecosystem services provision index and land development index to analyze ES supply and demand in China from 2000 to 2015 [14]. In a case study of Shanghai, Chen et al. found that there was a spatial mismatch between ES supply and demand, especially in urban centers [7]. In most of the research on ES supply and demand, the interrelationships between ES supply and demand have been emphasized. However, the relationship between multiple ES is complex and a combination of different approaches may more effectively explain the different characteristics of supply and demand. The matching relationship between ES supply and demand and the comprehensive assessment of various supply and demand situations require further detailed research [7,14,15]. At the same time, studies of ecological zoning management based on the supply and demand of ecosystem services are rare [10,16,17,18].

Located in the central region of China, Wuhan is an important node city in the Yangtze River Economic Belt, and one of the major cities undergoing rapid social and economic development in China. The rapid transformation in land use/land cover change (LUCC) in Wuhan has led to changes in the structure and interrelationships of ES supply and demand. Zhang et al. used the bivariate Moran’s I method to characterize the relationship between ecosystem services and urbanization in Wuhan, and observed a negative spatial correlation [19]. Luo et al. examined the effects of the “ecological control line” and its supporting policies on maintaining ecosystem services in Wuhan [20]. Wang et al. studied the impact of ecosystem services under different land-use scenarios [21]. Wuhan City therefore serves as a valuable research area for exploring the impacts of changes in LUCC on ES supply and demand.

The main objectives of the present study are: (1) to determine the spatial differentiation and evolution of ES supply and demand in Wuhan; (2) to apply the supply–demand ratio for individual ES and the comprehensive ES supply–demand ratio (CESDR) to assess the interrelationships between ES supply and demand; (3) to investigate ecological zoning based on CESDR; and (4) to produce land-use policy recommendations for the observed ecological zoning. Overall, it was hoped that the study would provide a decision-making reference for the sustainable utilization of urban land in Wuhan and elsewhere.

## 2. Materials and Methods

### 2.1. Study Area

**Figure 1:**
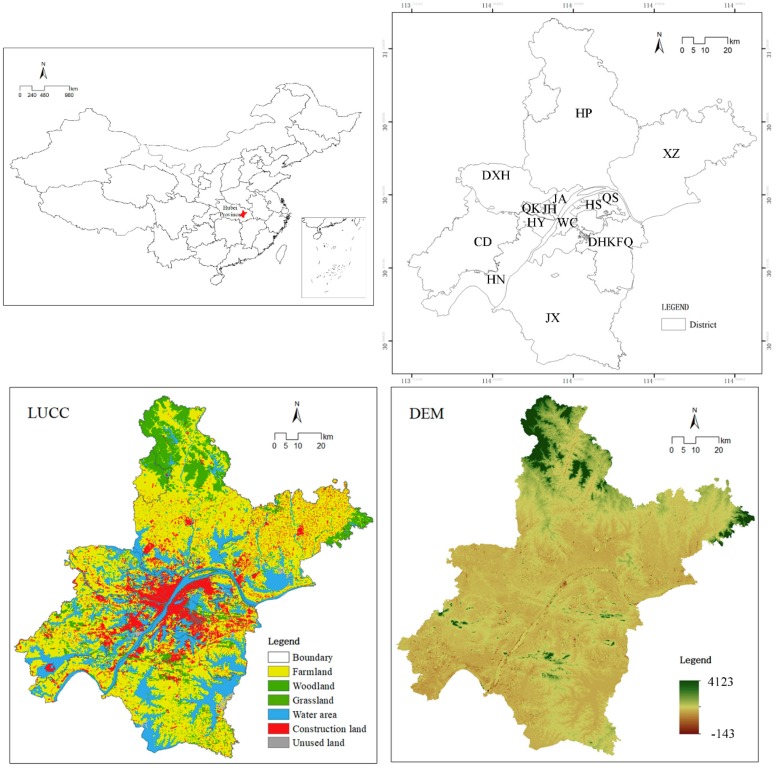
The study area of Wuhan City. Abbreviations: JX—Jiangxia, HS—Hongshan, WS—Wuchang, JA—Jiang’an, HP—Huangpi, CD—Caidian, JK—Jingkai, HY—Hanyang, QK—Qiaokou, JH—Jianghan, QS—Qingshan, XZ—Xinzhou, and DXH—Dongxihu.

Wuhan, the capital of Hubei Province, is located in the central part of the province where the Yangtze River merges with the Han River, hence water resources are abundant (Figure 1). The region experiences a subtropical monsoon climate. The city area is 8494.41 km^2^. In 2015 the residential population of Wuhan was 10.607 million, and the annual GDP was 109.5 billion RMB. As a result of the development of the Yangtze River Economic Belt and gradual advances in metropolitan area policy, Wuhan has become an important node city in central China.

The “Wuhan City Master Plan (1996–2020)” and “Wuhan City Master Plan (2010–2020)” were implemented with the aim of creating an attractive ecological environment. Since 2000, these plans have had a profound impact on land-use patterns and urban development in Wuhan.

### 2.2. Data Sources

LUCC data were obtained from the Resource and Environmental Science Data Center of the Chinese Academy of Sciences (http://www.resdc.cn/Default.aspx). The spatial resolution is 30 m. Land use is divided into six categories: Cultivated land, forest, grassland, open water, construction land, and unused land. The meteorological data were derived from the China Meteorological Data Network (http://data.cma.cn/), and includes temperature and precipitation in the study area and the surrounding eight stations. Spatial interpolation of the site data was used to obtain spatial raster data. The soil data, with a spatial resolution of 1 km, were from the World Soil Database (http://www.fao.org/land-water/databases-and-software/hwsd/en/). They include soil depth, organic matter content, bulk density, and the contents of sand, silt, and clay. The DEM data were downloaded from the geospatial data cloud (http://www.gscloud.cn/), and have a spatial resolution of 30 m. In ArcGIS, the vector boundary of the watershed/secondary watershed was extracted using the ArcSWAT plugin. The Normalized Difference Vegetation Index (NDVI) was generated from Moderate Resolution Imaging Spectroradiometer (MODIS) images, with a resolution of 250 m, for 2000–2015 (http://www.resdc.cn/Default.aspx). Data on grain yield, energy consumption, and food consumption were from the statistical yearbooks of China, Hubei Province, and Wuhan (http://www.statshb.gov.cn/info/iIndex.jsp?cat_id=10055; http://www.stats.gov.cn/tjsj/ndsj/). The water consumption data were from the water resources bulletin of Hubei Province (http://www.stats-hb.gov.cn/info/iIndex.jsp?cat_id=100) and Wuhan City (http://www.stats.gov.cn/tjsj/ndsj/). The per capita public green land index data refer to the city of Wuhan. The data of per capita public green space index refer to the requirements specified in the Wuhan City Master Plan (http://gtghj.wuhan.gov.cn/). Data used to quantify ES supply and demand in Wuhan are list in Table S1.

### 2.3. Mapping the Supply and Demand for Four Ecosystems Services

Based on their importance in Wuhan and on data availability, four ES indicators were used: Water yield and grain yield (both from the provisioning services), climate regulation (from the regulating services) and recreation services (from the cultural services). Data for 2000, 2010, and 2015 were obtained. Also, Supplementary Table S2 list key parameters used to quantify ecosystem services (ES) supply and demand in Wuhan. 

#### 2.3.1. Water Yield (WY)

(1) Supply of water yield (S_WY_)

Water retention is defined as the amount of water resources available to human beings, equal to the difference between precipitation and actual evapotranspiration and environmental flow demand [8]. It is calculated as

(1) $$S_{WY}=\mathrm{Pre}-{\mathrm{ET}}_{actual}-EF$$

where $S_{WY}$ is the supply of water yield service, Pre is annual precipitation, ${\mathrm{ET}}_{actual}$ is actual evapotranspiration, and EF is the demand for the environmental flow rate. Based on the principles of water balance, the water production module in the InVEST model was used for the calculation [22].

(2) Demand for water yield (D_WY_)

D_WY_ refers mainly to urban and rural domestic water, agricultural water, industrial water and ecological water, including for humans [7,8]. It is calculated as

(2) $$D_{WY}=D_{urban\;and\;rural}+D_{agr}+D_{in}+D_{eco}$$

where $D_{WY}$ is the demand for water resources, $D_{urban\;and\;rural}$ is the urban and rural domestic water demand, $D_{agr}$ is the agricultural water demand, $D_{in}$ is the industrial water demand, and $D_{eco}$ is the ecological water demand.

#### 2.3.2. Grain Yield (GY)

(1) Supply of grain yield (S_GY_)

S_GY_ is calculated according to the output per unit area of various crops (including grains, beans, and root crops) [23]. Various studies have shown that there is a significant linear relationship between grain yield and NDVI [24], and therefore NDVI data were used to spatialize grain yields; that is, grain yield were allocated to cultivated land grids according to NDVI values, and then used to calculate the grain yield. It is calculated as follows:

(3) $$G_{i}=\frac{{\mathrm{NDVI}}_{i}}{{\mathrm{NDVI}}_{sum}}\times G_{sum}$$

(4) $$S_{GY}=G_{i}/Area$$

where $G_{i}\;is$ the grain yield allocated for grid i, $G_{sum}$ is the total food production, ${\mathrm{NDVI}}_{i}$ is the NDVI of grid i, ${\mathrm{NDVI}}_{sum}$ is the sum of NDVI of the cultivated land in the study area, $S_{GY}$ is the grain yield per unit area of grid i (that is, the supply of grain yield), and Area is a single grid area.

(2) Demand of grain yield (D_GY_)

D_GY_ is expressed in terms of grain consumption per unit area. The grain consumption of urban and rural residents is calculated based on population density and per capita grain consumption of urban and rural residents [25]. It is calculated as

(5) $$D_{GY}=Den_{pop}\times Per_{u}+Den_{pop}\times Per_{r}$$

where $D_{GY}$ is the demand of grain yield, $Den_{pop}$ is the population density, $Per_{u}$ is the per capita grain consumption of the city, and $Per_{r}$ is the per capita grain consumption in rural areas. The product of $Den_{pop}$ and $Per_{u}$ is the grain yield demand of urban residents, and the product of $Den_{pop}$ and $Per_{r}$ is the grain yield demand of rural residents.

#### 2.3.3. Climate Regulation (CR)

(1) Supply of climate regulation (S_CR_)

The climate can be adjusted by increasing or decreasing the concentration of atmospheric greenhouse gases such as carbon dioxide. It is calculated using the carbon storage module in the InVEST model. For each LUCC type, the module needs to estimate the minimum amount of carbon in each of the four main carbon pools (aboveground, underground, soil, and dead organic material) [22]. It is calculated as

(6) $$S_{CR}=C_{above}+C_{below}+C_{soil}+C_{dead}$$

where $S_{CR}$ is the total carbon reserves, $C_{above}$ is the aboveground carbon stock, $C_{below}$ is the underground carbon stock, $C_{soil}$ is the soil carbon stock, and $C_{dead}$ is the carbon stock of dead organic matter. The carbon storage table in the basic carbon pool of the LUCC unit was obtained by consulting the IPCC appendix of the InVEST user manual [22] together with the actual LUCC classification in Wuhan.

(2) Demand of climate regulation (D_CR_)

The main sources of carbon outflows can be divided into industrial activities (e.g., raw coal, washed coal, coke, crude oil, fuel oil, gasoline, diesel oil, kerosene, refinery dry gas, liquefied petroleum gas, coke oven gas, etc.) and agricultural activities (e.g., fertilizers, pesticides, agricultural film, or agricultural diesel oil) [26]. Thus, carbon outflows can be calculated as

(7) $$D_{CR}=\sum D_{i}=\sum C_{i}\times b_{i}$$

where $D_{CR}$ is the total amount of carbon outflows, $D_{i}$ is the amount of carbon outflows from various carbon sources, $C_{i}$ is the amount of each carbon source, and $b_{i}\;is$ the carbon emission coefficient of each carbon source.

#### 2.3.4. Recreation Services (RS)

(1) Supply of recreation services (S_RS_)

Public green space is an important provider of outdoor recreation and leisure. S_RS_ can be evaluated from the proportion of public green space area relative to the total area [27]. S_RS_ in a specific area (streets, towns, etc.) can be expressed by the ratio of the area of green space (woodland or grassland) to the total area, as follows:

(8) $$S_{RS}=G_{i}/Z_{i}$$

where $S_{RS}$ is the supply of recreation and leisure (that is, the green space rate of region i), $G_{i}$ is the area of green space within region i, and $Z_{i}$ is the area of region i.

(2) Demand for recreation services (D_RS_)

D_RS_ is mainly determined by the per capita green space index determined by population density and government planning [7]. It is calculated as

(9) $$D_{RS}=Den\times P$$

where $D_{RS}$ is the demand for recreation and leisure, Den is the population density, and P is the per capita green space index.

### 2.4. Construction and Evaluation of the ES Supply and Demand Index

Constructing an index of the supply–demand ratio helps to demonstrate visually the dynamic spatio-temporal characteristics of the equilibrium relationship between ES supply and demand. According to the supply–demand ratio defined by Chen et al. (2019) [7], the ES supply–demand ratio and comprehensive ES supply–demand ratio in Wuhan were calculated as follows.

#### 2.4.1. ES Supply–Demand Ratio (ESDR)

The ES supply–demand ratio links the actual supply of ES to human needs and can be used to reveal surpluses or shortages of ecosystem services [7,28]. It is calculated as

(10) $$ESDR_{i}=\frac{S_{i}-D_{i}}{\left(S_{max}+D_{max}\right)/2}$$

where $S_{i}$ and $D_{i}$ are the actual ES supply and demand for ES type I, and $S_{max}$ and $D_{max}$ are the maximum of ES supply and demand, respectively. A positive value indicates an ES surplus, zero indicates a balance between ES supply and demand, and a negative value indicates a shortage of ES supply relative to demand.

#### 2.4.2. Comprehensive Supply–Demand Ratio (CESDR)

The Comprehensive supply–demand ratio (CESDR) is used to determine the state of ES at an integrated level. It is calculated as the arithmetic mean of the ESDR:

(11) $$\mathrm{CESDR}=\frac{1}{n}\overset{n}{\underset{i=1}{\sum }}ESDR_{i}$$

where *n* = 4, and ESDR is the value of the supply–demand ratio for each type of ecosystem service.

We also quantified changes in the gradient in CESDR along four sample transects as a function of distance from the urban center in response to changes in urban land use and land cover change.

## 3. Results

### 3.1. Evolution of Relationships Between ES Supply and Demand

#### 3.1.1. Water Yield 

**Figure 2:**
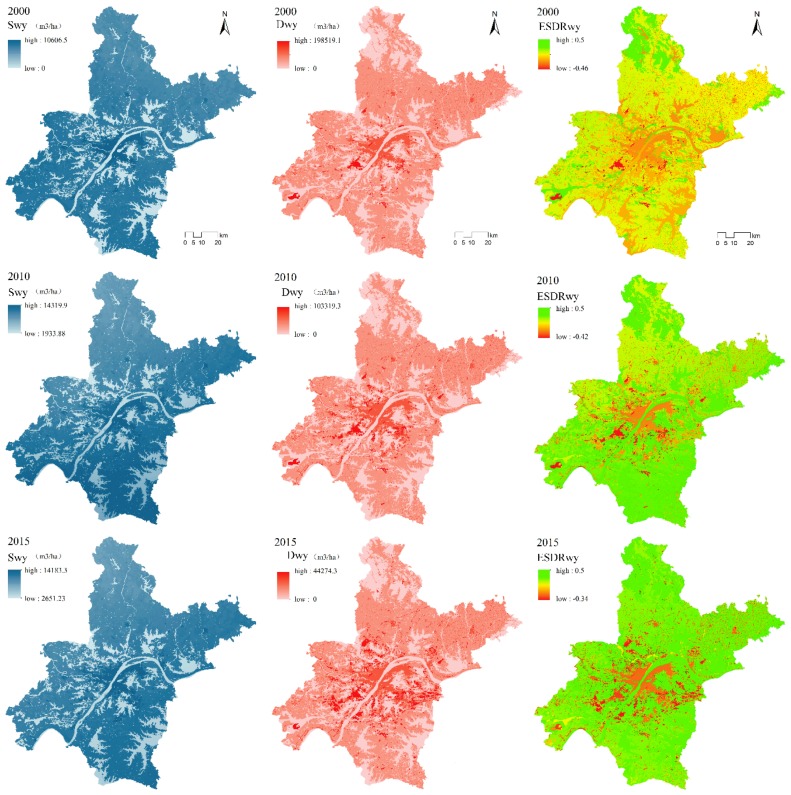
Spatial distribution of supply, demand, and supply–demand ratio of water yield service in Wuhan in 2000, 2010, and 2015.

Figure 2 shows that during 2000–2015, the water yield supply (S_WY_) and water yield demand (D_WY_) in Wuhan City showed an upward trend. In particular, the water yield supply in 2000 (S_WR2000_) in Wuhan was 6752.25 m^3^/ha; in 2010 (S_WY2010_) it was 10,069.88 m^3^/ha; and in 2015 (S_WY2015_) it was 9820.75 m^3^/ha. The total amount of S_WY_ in Wuhan during 2000–2015 increased from 5.876 billion m^3^ in 2000, to 8.622 billion m^3^ in 2010, and to 8.409 billion m^3^ in 2015. In addition, the water yield demand in 2000 (D_WY2000_) was 4.233 billion m^3^; in 2010 (D_WY2010_) it was 3.933 billion m^3^; and in 2015 (D_WY2015_) it was 3.759 billion m^3^.

Comparison of the spatial distribution of S_WY_ with D_WY_ reveals that areas of high/low S_WY_ correspond roughly to areas of high/low D_WY_, indicating that S_WY_ is matched by D_WY_. In 2000, the range of ESDR_WY_ was −0.46 to 0.5; in 2010 it was −0.42 to 0.5; and in 2015 it was −0.34 to 0.5. The spatial relationship between S_WY_ and D_WY_ in 2010 and 2015 has changed compared to the relationship in 2000, mainly because S_WY_ and D_WY_ in the central urban area have gradually changed from a state of relative balance to one of scarcity, and the range has also changed with the expansion of central Wuhan. On the other hand, a state of relative equilibrium in the southeastern part of the city in 2000 gradually evolved to one of relative surplus in 2010 and 2015.

#### 3.1.2. Grain Yield 

**Figure 3:**
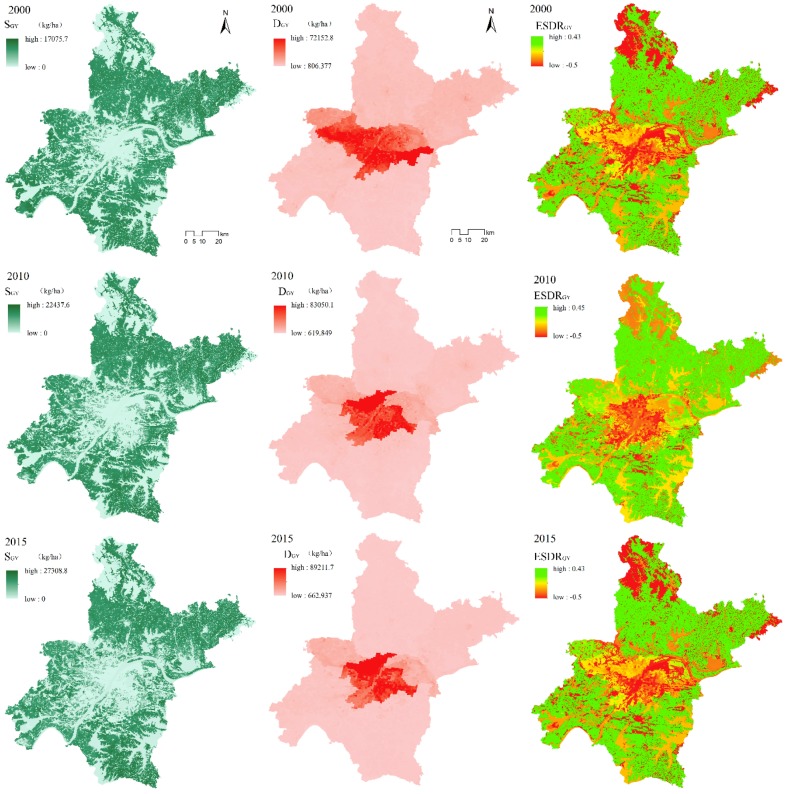
Spatial distribution of supply, demand, and supply–demand ratio of grain yield service in Wuhan in 2000, 2010, and 2015.

The total grain yield supply values (S_GY_) for Wuhan in 2000, 2010, and 2015 were 56 Mt, 71.9 Mt, and 82.9 Mt, respectively. In 2000, the average S_GY_ was 6995.26 kg/ha; in 2010 it was 8753.35 kg/ha; and in 2015 it was 10,075.77 kg/ha. The average grain yield demand (D_GY_) in Wuhan in 2000 was 4227.52 kg/ha; in 2010 it was 3341.83 kg/ha; and in 2015 it was 3345.58 kg/ha. In 2015, D_GY_ was 880.94 kg less than in 2000, which indicates a reduction in D_GY_ per unit area in Figure 3.

Comparison of the average values of S_GY_ and D_GY_ in Wuhan indicates that S_GY_ is greater than D_GY_. Figure 3 shows that areas with larger values of D_GY_ correspond to areas with lower values of S_GY_, and vice versa. Thus, there is a mismatch between S_GY_ and D_GY_, and thus an imbalance between S_GY_ and D_GY_.

The range of the grain yield supply–demand ratio (ESDR_GY_) is from −0.5 to 0.46. It can be seen that the difference between S_GY_ and D_GY_ in Wuhan is substantial. During 2000–2015, ESDR_GY_ gradually decreased from 0.46 to 0.43, while the negative value remained at 0.5. It can be seen that the state of grain yield service surplus is approaching equilibrium, and that a shortage still occurs in D_GY_.

In terms of the spatial distribution of ESDR_GY_, the range is negative. It includes two major areas: The urban center, and the forested land in the northwest part of the city. The (negative) value of the urban central area has continued to decrease over time. The range of the central red region has gradually expanded from the east bank of the Yangtze River to the west bank, and then to the entire central area. At the same time, the distribution has changed from being relatively fragmented to continuous, while the overall state of the northwest area has improved, as shown by the red shading in Figure 3. ESDR_GY_ tends to zero; that is, areas where S_GR_ and D_GR_ are roughly in balance (the yellow shaded area in Figure 3) are distributed mainly in the suburbs outside the central city. There are many patches with a deficit embedded within the southern part of the city; the number of these patches has decreased during 2000–2015.

#### 3.1.3. Climate Regulation 

**Figure 4:**
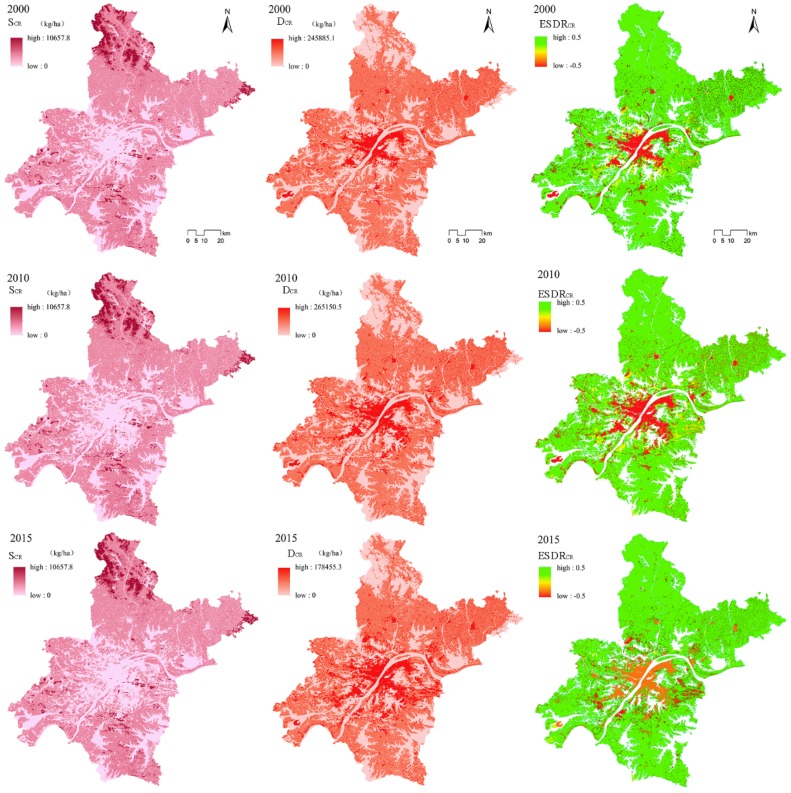
Spatial distribution of supply, demand, and supply–demand ratio of climate regulation service in Wuhan in 2000, 2010, and 2015.

The total carbon storage in Wuhan in 2000 (S_CR2000_) was ~0.190 million ton; in 2010 (S_CR2010_) it was ~0.184 million ton; and in 2015 (S_CR2015_) it was ~1.79 million ton. Thus, S_CR_ in Wuhan decreased continuously during 2000–2015. The average carbon outflows in Wuhan in 2000 (D_CR2000_) were 19.08 kg/ha; in 2010 (D_CR2010)_ they were 26.33 kg/ha; and in 2015 (D_CR2015_) they were 22.21 kg/ha. Thus, during 2000–2015, D_CR_ first increased and then decreased. The region with the highest S_CS_ has the lowest D_CR_, and therefore S_CR_ and D_CR_ are spatially mismatched in Figure 4.

ESDR_CR_ ranges from −0.5 to +0.5, indicating substantial regional differences in S_CR_ and D_CR_. From the spatial distribution of EDSR_CR_, the area with surplus S_CR_ and D_CR_ is larger than the area with a scarcity. In 2000 and 2010 the distribution of areas of scarcity had the form of “1 + N”, where 1 represents the central city, and N represents several patches in the city (Figure 4). However, in 2015 the relationship between S_CR_ and D_CR_ in the central urban area trended to be more balanced (in Figure 4, it can be seen that the color of the central part of the city changed from red to orange). In 2000, the relationship between S_CR_ and D_CR_ (the yellow shaded area in Figure 4) was more balanced and distributed to the north and south of the central area. In 2010, the area with an approximate balance increased substantially and also had a circular distribution around the central area. In 2015, the area with a balance between S_CR_ and D_CR_ decreased substantially, while the area of surplus was mainly in the outer suburbs.

#### 3.1.4. Recreation Services

**Figure 5:**
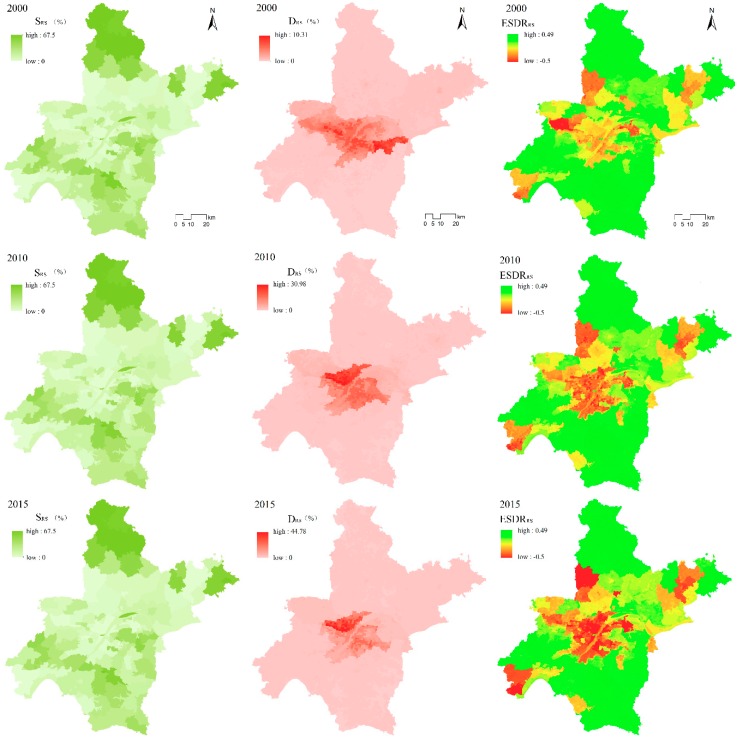
Spatial distribution of supply, demand, and supply–demand ratio of regulation services in Wuhan in 2000, 2010, and 2015.

In 2000, 2010, and 2015 the sum of S_RS_ was larger than that of D_RS_ (Figure 5), and during this interval D_RS_ increased and S_RS_ decreased. Comparison of S_RS_ and D_RS_ in Figure 5 reveals a spatial mismatch_._ In the area to the south of the Yangtze River, in central Wuhan, S_RS_ is relatively low, but there was a large demand for a high green space ratio of the towns in this area, and therefore it was difficult for S_RS_ to satisfy the D_RS_. In contrast, in the northwestern, northeastern, and southern suburbs of the city, S_RS_ was relatively high in Figure 5.

Analysis of the ESDR of ES_RS_ for 2000 reveals that the surplus area of ES_RS_ in Wuhan was the largest. In 2010, the area of balance had decreased sharply, with the area of shortage substantially increased, and with a large area (mainly in the urban center) changing from a state of approximate balance to a shortage of S_RS_ which could not meet D_RS_. In 2015, the area of balance had changed to an area of shortage. If this trend continues, the shortage will be further exacerbated in areas that already have a short supply.

### 3.2. Mismatches between Comprehensive ES Supply and Demand

#### 3.2.1. Mapping the Comprehensive ES Supply and Demand Ratio

**Figure 6:**
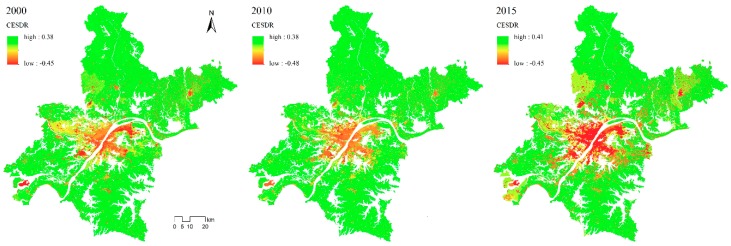
Spatial distribution of the comprehensive ecosystem services supply–demand ratio (CESDR) from 2000 to 2015.

The CESDR, as defined above, was used to analyze the relationship between the integrated ecosystem services supply and demand in Wuhan. From Figure 6, it can be seen that:

(1) There is substantial spatial heterogeneity of the supply and demand relationship of ecosystem services in Wuhan. The comprehensive supply and demand index has a large range, with a maximum difference of 0.86.

(2) There is a large spatial mismatch between ES supply and demand. For the central part of the city, CESDR is negative and the absolute value is large, and thus the ES supply does not meet the demand. In contrast, in the outer suburbs, the CESDR is positive and the absolute value is large, and the ES supply is greater than the demand.

(3) The spatial relationship between ES supply and demand in Wuhan City has evolved to a circular distribution. There is a deficit in ES in the central urban area, supply and demand in the suburbs are approximately in balance, and supply is greater than demand in rural areas.

(4) The imbalance between ES supply and demand in Wuhan has intensified over time. Quantitatively, the difference between the comprehensive supply and demand index is increasing with time, the ecosystem service surplus area is continuing to grow, and the area of shortage is increasing due to rising demand, resulting in a growing gap between supply and demand. Over time, the areas where ecosystem services are scarce have expanded at the expense of the areas with an approximate balance, while the areas with an approximate balance have extended to areas with a surplus.

#### 3.2.2. Changes in Comprehensive ES Supply and Demand Along Different Directions within Wuhan

**Figure 7:**
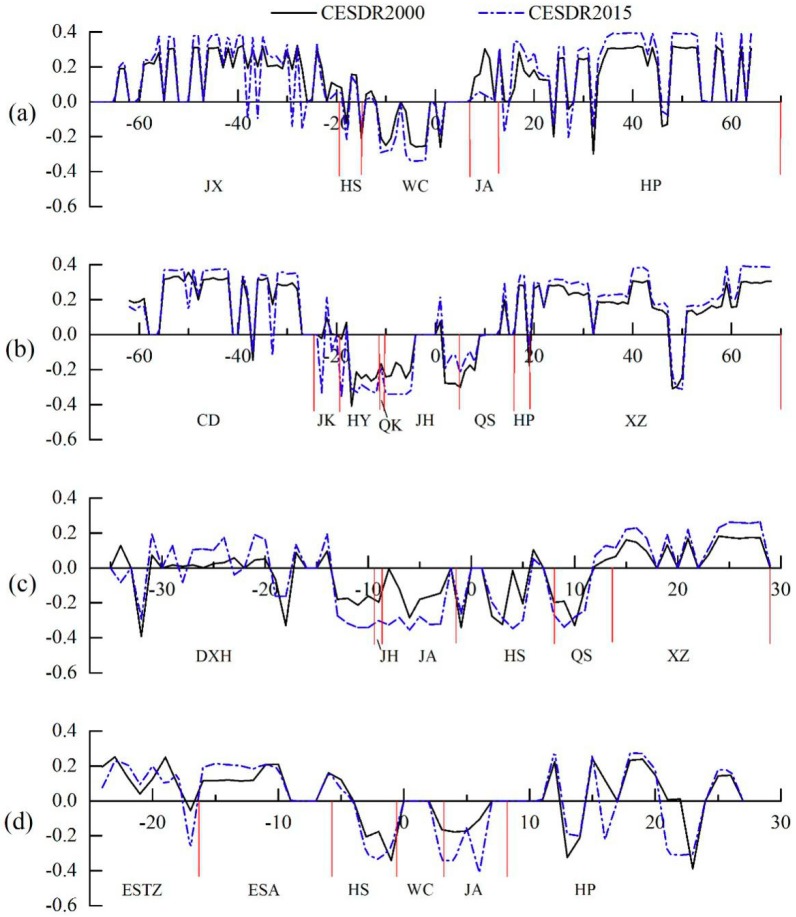
Results of gradient analysis of CESDR along four transects: (**a**) north–south, (**b**) east–west, (**c**) northeast–southwest, and (**d**) northwest–southeast in Wuhan City.

We attempted to characterize spatial gradients in the ES supply and demand in Wuhan over the past 15 years. This was done by taking the central point of Wuhan and analyzing the supply–demand ratio of integrated ecosystem services along zones of 1 km wide outwards along four directions (W–E, S–N, SW–NE, and NW–SE), which represent axes of urban development [26,27,28]. There are substantial differences in the ES supply of and demand along these axes. In the central urban area, there is a shortage of ES (CESDR < 0), while at distances far from the urban central areas there is an oversupply (CESDR > 0). Along the S–N axis (Figure 7a), CESDR values in Jiangxia and Huangpi districts in the outer suburbs are higher, while those in Wuchang District in the central urban districts are mostly negative. Along the SW–NE axis (Figure 7b), Caidian, Huangpi, and Xinzhou are mainly cultivated land and woodland and ecosystem services are in surplus, while in Hanyang, Qiaokou, and Jianghan they are in deficit. The ES supply and demand in Qingshan District, located in the urban–rural intersection, changes from deficit to surplus with increasing distance from the urban center. Along the E–W axis (Figure 7c), ecosystem services in Jianghan, Jiangan, and Hongshan districts in the urban center are in deficit (CESDR < 0), while in East–West Lake and Xinzhou, in the suburbs, they are in surplus (CESDR > 0). Similarly, ecosystem services in Qingshan District change from deficit to surplus with increasing distance from the urban center. Along the NW–SE axis (Figure 7d), ecosystem services in Hongshan, Wuchang, and Jiangbian districts, in the central urban area, are in deficit. Most of the ecosystem services in the Donghu High-tech Development Zone, Donghu Scenic Area, and Huangpi, which have a greater area of woodland and arable land, are in surplus. Comparison of the change in the ratio of integrated ecosystem services between 2000 and 2015 reveals that the regional deficit of ES supply and demand for Wuhan became more pronounced in the urban center, especially in the riverside area (Figure 7c,d). However, in the suburbs, there was only a minor change in ES supply and demand.

### 3.3. Ecological Zoning Management

**Figure 8:**
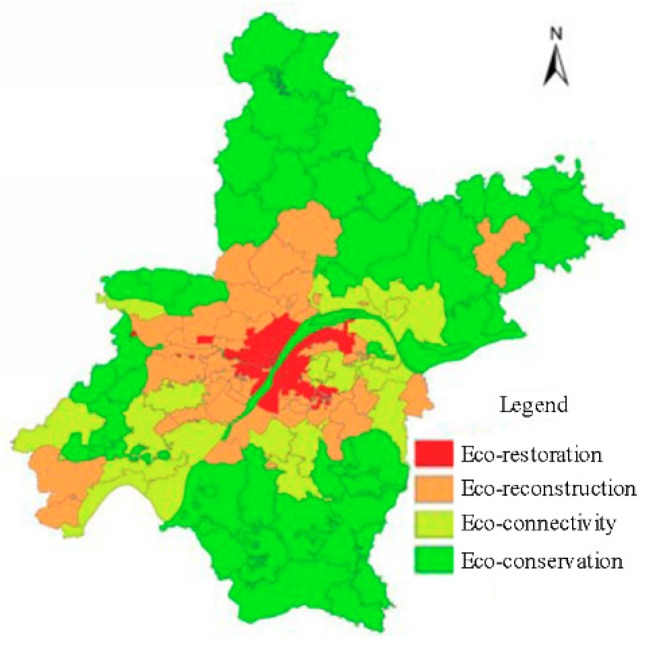
Distribution of ecological construction zones in Wuhan.

The foregoing analysis has revealed substantial spatial differences in supply–demand relationships for ES in Wuhan. In order to provide a rational basis for the development of an ecological civilization and to promote sound ecological management, it is necessary to carry out a policy of ecological regionalization and differential management. Based on the spatial differentiation of the comprehensive supply–demand index of ecosystem services in 2015, the ecological zonation of Wuhan can be divided into four types: Ecological restoration, ecological reconstruction, ecological connectivity, and ecological conservation. They correspond to four supply–demand relationships: Low supply and high demand, low supply and low demand, high supply and low demand, and high supply and high demand. Combined with the map of ecological zonation construction (Figure 8), we propose the following recommendations for ecological construction:

(1) Eco-restoration zones

These comprise 75 streets/townships which are concentrated in the urban center. Construction land is the main land use in the region. The degree of land development and utilization is high, the population is dense, ecological patches are few in number and it is difficult for the local ecological needs to be met. In terms of management, first, the degree of protection of ecological land should be strengthened, and second, ecological construction should be increased, making full use of the existing patchwork of ecological spaces, and the coverage of urban green space should be increased. In addition, the land utilization ratio should be improved, the urban land should be rejuvenated, and strict controls should be implemented to prevent urban construction from encroaching on ecological land.

(2) Eco-reconstruction zones

These comprise 43 streets/townships, distributed in the form of a “C-shape” around the city’s central area. The land use in this area is complex and represents an expansion area of the city. Both the intensity of land use and the population are increasing continuously, and the areas of ecological land are seriously threatened. At present, however, it is able to meet its own ecological needs. It is necessary to integrate and promote the existing ecological resources, strengthen ecological protection and restoration, improve land use efficiency, and construct a complex social–ecological–economic space.

(3) Eco-connectivity zones

These include 20 streets/townships, distributed in the central-east and southwest parts of the city. Cultivated land and open water are the main types of land use, the intensity of land development is relatively low, the economic base is weak, and the ecological demand is low. Therefore, an eco-economy can be developed, including promoting local industrial restructuring and linking urban and rural areas with the eco-economy, in order to alleviate the high ecological needs of the urban population. Such a policy would also promote the rural economy and provide substantial ecological and economic benefits.

(4) Eco-conservation zones

These comprise 48 streets/townships, mainly distributed in the north and southeast parts of the city. This region is dominated by woodland, arable land, and open water, where the natural resources are more than sufficient to meet its own ecological needs. The region is the source of the flow of ecosystem services to other regions and it is important to protect urban green space, water systems and cultivated land, promote the role of ecological barriers and lakes, maximize water conservation, and enhance the supply of raw materials from cultivated land, in order to ensure the sustainable delivery of ecosystem services to the surrounding areas.

## 4. Discussion

### 4.1. Trade-Off and Synergy of Ecosystem Services Supply

**Figure 9:**
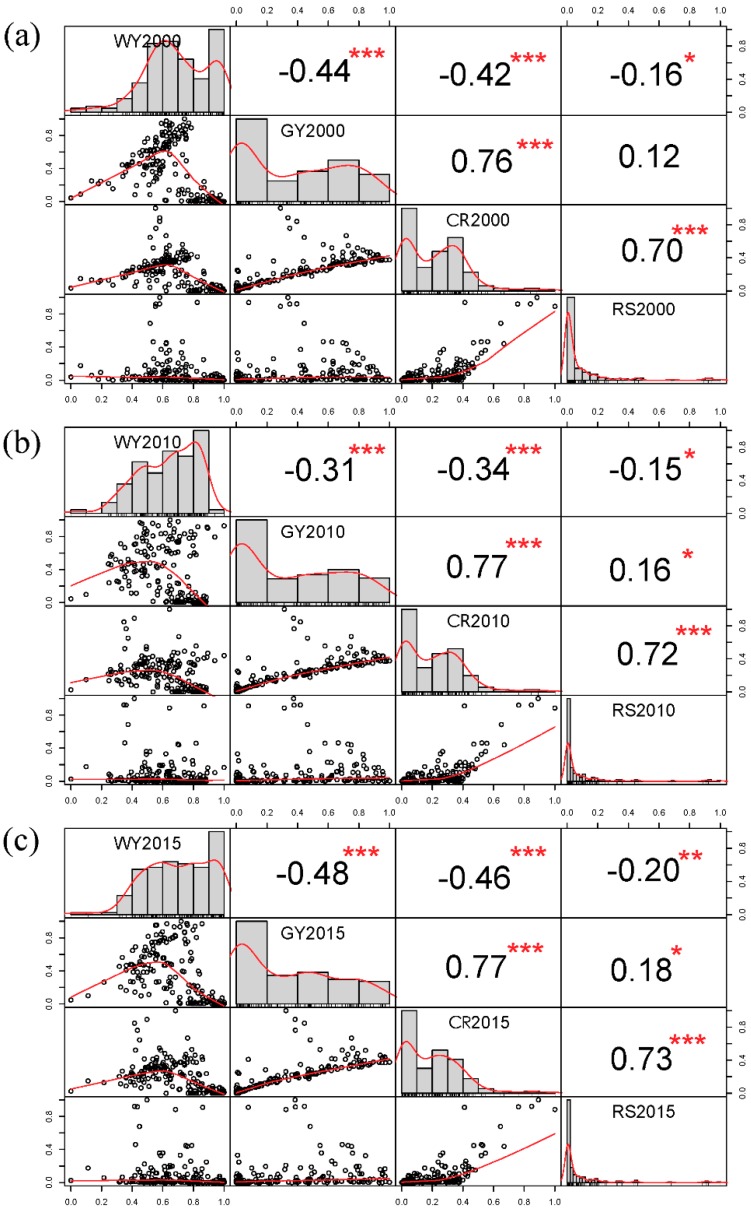
Matrix of Pearson’s correlation coefficients between the four ES in 2000 (**a**), 2010 (**b**), and 2015 (**c**). * *p* < 0.1; ** *p* < 0.05; and *** *p* < 0.01.

On the city scale, the statistical method of districts was used to quantify the supply of various ecosystem services, and the calculation results were standardized in Figure 9. Correlation analysis was used to evaluate the relationship between the supply of four ecosystem services for Wuhan. It was found that there is a significant trade-off relationship between the S_WY_ and S_GY_ in Wuhan. The correlation coefficient between the two is −0.44 (*p* < 0.01) for 2000, −0.31 (*p* < 0.01) for 2010, and −0.48 (*p* < 0.01) for 2015. There is also a significant trade-off between the S_WY_ and the S_CR_. The correlation coefficients between the two are −0.42 (*p* < 0.01) for 2000, −0.34 (*p* < 0.01) for 2005, and −0.46 (*p* < 0.01) for 2015. These findings are similar to those of Zhang [19] for Wuhan. Attention should be paid not only to the correlation between the ES supply and demand, but also to the interrelationships among the different supplies of ecosystem services.

### 4.2. Practical Implications of the Imbalance Between ES Supply and Demand

In this research, ESDR and CESDR were used to depict the relationship between ES supply and demand, which represented a spatial pattern of the match degree of the production of ES and services needs of human living. It was found that the imbalance between supply and demand is caused by the difference between supply and demand [29]. Especially in the rapidly expanding areas of the city, the ES supply has experienced significant degradation. At the same time, the ES demand has increased significantly, resulting in a shift from a balance to an imbalance in the ES supply and demand in the region, for example, in the urban and rural areas of Wuhan. Multi-functional rural landscapes can provide more ES supply to humans and thus make an important contribution to human well-being, and adding or optimizing green infrastructure in urban areas will improve the supply of ES [30]. For most provisioning services, the imbalance between supply and demand appears to be easier to resolve. The imbalance between supply and demand for most services appears to be easier to resolve, for example, the problem of local S_GY_ not meeting the D_GY_ can be solved by the allocation of grain between regions [31].

Although spatial explicit models may be one of the most common models of ES supply and demand, their application still has several limitations in the assessment process. Due to the complexity of ES, we have simplified them when using models for evaluation. For example, when assessing the supply of climate regulation in Section 2.3.3, we have characterized it as “carbon sequestration” and evaluated it using the carbon storage module in the InVEST. From Equation (6), we can see that the premise of this method is that the carbon storage coefficient of the same land-use type is the same, and it will not increase or decrease carbon over time, or under the influence of climate change. To address this problem, future research could increase the level of detail of land-use changes, and classify some LUCC types according to the length of time [32]. In addition, most of the data we use is annual data, but the annual variance in the calculated indicators may have an impact on the ES supply and demand, such as the estimated annual precipitation of water production in this study, while the regional precipitation is obviously subject to seasonal changes which will affect the accuracy of the assessment of ES.

## 5. Conclusions

Land use changes and the related development of Wuhan City have had varying ecological impacts. In this study we have used Wuhan as a case study to assess the supply of and demand for ecosystem services, specifically, water resources, food production, climate regulation, and leisure and entertainment provision. The spatio-temporal distribution of the four types of service and their spatial relationship provide decision support for the sustainable development of regional land use. We used the two indicators of ESDR and CESDR to measure the matching and agglomeration of ES supply and demand in the city. Based on the results of CESDR analysis, an ecological subdivision of Wuhan City is proposed. Various zoning policy recommendations provide a decision-making reference for sustainable land development in the region. Our main findings are as follows:

(1) From the perspective of total supply and demand, the supplies of water yield, grain yield, and recreation services are greater than the demand, and the supply of climate regulation is less than the demand. In addition, there are spatial imbalances and mismatches in the supply of and demand for various ecosystem services, especially in the urban central areas. Notably, the area of imbalance has expanded with the gradual increase in the built-up area.

(2) The ecological subdivision of the comprehensive ecosystem service supply and demand ratio exhibit a circular distribution in the form of “stable area–relative equilibrium area–surplus area”, which corresponds to “urban central area–near suburbs–distant suburbs and rural area”. According to the ecological zonation characteristics, a policy of sustainable land use is proposed in the context of “ecological conservation–ecological restoration–ecological reconstruction–ecological connectivity”.

## Supplementary Materials

The following are available online at https://www.mdpi.com/1660-4601/16/13/2332/s1, Table S1: Data used to quantify ecosystem services (ES) supply and demand in Wuhan; Table S2: Key parameters used to quantify ecosystem services (ES) supply and demand in Wuhan.
