# Supplementary material for: Evaluating Ecosystem Services Supply and Demand Dynamics and Ecological Zoning Management in Wuhan, China
Source: Int J Environ Res Public Health. 2019 Jul 2;16(13):2332. doi: 10.3390/ijerph16132332 (PMC6651407; doi:10.3390/ijerph16132332)
Supplement: Supplementary file 1 [file ijerph-16-02332-s001.pdf]

International Journal of
Environmental Research and Public Health

Supplementary Materials

Evaluating Ecosystem Services Supply and Demand Dynamics and Ecological Zoning Management in Wuhan, China

Feiyan Chen [1,2,3], Ling Li [1] , Jiqiang Niu [2,3], Aiwen Lin [1,*], Shiyu Chen [2,3] and Lin Hao [2,3]

1 School of Resource and Environmental Science, Wuhan University, Wuhan 430079, China; feiyanchen16@126.com (F.C.); liling210@whu.edu.cn (L.L.)
2 School of Geographic Sciences, Xinyang Normal University, Xinyang 464000, China; niujiqiang@xynu.edu.cn (J.N.); csy_hy@whu.edu.cn (S.C.); hlin-rs@whu.edu.cn (H.L.)
3 Key Laboratory for Synergistic Prevention of Water and Soil Environmental Pollution, Xinyang Normal University, Xinyang 464000, China;
* Correspondence: awlin@whu.edu.cn; Tel.: +86-152-9474-8826

Table S1. Data used to quantify ecosystem services (ES) supply and demand in Wuhan.

Data	Data description	Spatial resolution	Publisher/source	Data source/reference
	DEM	30 m		http://www.gscloud.cn/
	Basin boundary			http://www.gscloud.cn/
	Administrative boundary		Chinese Academy of Sciences	http://www.resdc.cn/Default.aspx
Land use	LUCC	100 m	Resource and Environmental Science Data Center	http://www.resdc.cn/Default.aspx
Climate	Precipitation	Site data	the China Meteorological Data Network	http://data.cma.cn/
Soil data	The biggest root soil buried depth	1000	the World Soil Database	http://www.fao.org/land-water/databases-and-software/hwsd/en/
	Available plant water		the World Soil Database/Gupta and Larson's empirical formula	Gupta S, Larson W. [1]
Other raster data	NDVI	1000	Chinese Academy of Sciences	
	population density	1000	Resource and Environmental Science Data Center	http://www.resdc.cn/Default.aspx
	Evapotranspiration (ETP)	1000	Global drought and potential evapotranspiration database	http://www.cgiar-csi.org/data/global-aridity-and-pet-databas； http://WorldCHm.or
Social and economic statistical data	Grain yield<br>Energy consumption data<br>Grain consumption per person		the statistical yearbooks of China, Hubei Province and Wuhan	http://www.stats-hb.gov.cn/info/iIndex.jsp?cat_id=10055; http://www.stats.gov.cn/tjsj/ndsj/
	Water consumption		Wuhan water resources bulletin, Hubei water resources bulletin	http://www.hubeiwater.gov.cn/szy/list.aspx?tid=271&page=1
Planning indicators	Planned green space index		Wuhan City Master Plan	http://gtghj.wuhan.gov.cn/
Related reference coefficient	table of department of biophysics<br>Carbon pool		InVEST Model User Manual	https://naturalcapitalproject.stanford.edu/invest/
	Carbon emission coefficient			Tian et al. [2]
	Seasonal constant			Ke xinli et al. [3]

Table S2. Key parameters used to quantify ecosystem services (ES) supply and demand in Wuhan.

**ES**	**Specific ES**	**Supply and Demand**	**Indicators**	**Data used**	**Comments**
Provisioning services	Water yield (WY)	S_{WY}	Water retention	Precipitation, LULC, Available plant water, Evapotranspiration (ETP), Available plant water, the biggest root soil buried depth, Table of department of biophysics, Seasonal constant	Formula (1) in Section 2.3.1
		D_{WY}	Water consumption	urban and rural domestic water, agricultural water, industrial water and ecological water	Formula (2) in Section 2.3.1
	Grain yield (GY)	S_{GY}	Grain production	Grain production, NDVI	Formula (3) and (4) in Section 2.3.2
		D_{GY}	Grain consumption	Grain consumption per person, population density	Formula (5) in Section 2.3.2
Regulating services	Climate regulation (CR)	S_{CR}	Carbon sequestration	LULC, Carbon pool	Formula (6) in Section 2.3.3
		D_{CR}	Carbon emission	Carbon emission coefficient	Formula (7) in Section 2.3.3
Cultural services	Recreation services (RS)	S_{RS}	Green space rate	LULC, Administrative boundary	Formula (8) in Section 2.3.4
		D_{RS}	Planned green space index	Planned green space index, population density	Formula (9) in Section 2.3.4

References

1. Gupta, S.C.; Larson, W.E. Estimating soil water retention characteristics from particle size distribution, organic matter percent, and bulk density. *Water Resour. Res.* **1979**, *15*, 1633–1635.
2. Tian, Y.; Li, B. Research on Measurement and Effecting Factors Decomposition of Carbon Emission in Wuhan. *Areal Res. Dev.* **2011**, *30*, 88–92.
3. Ke, X.; Pu, K.; Yang, B.; Zheng, W. Impact of cultivated Land protection on water petention function of ecosystem. *Res. Soil Water Conserv.* **2018**, *25*, 391–396.
